# Effective carrier-free gene-silencing activity of cholesterol-modified siRNAs†

**DOI:** 10.1039/c8ra03908a

**Published:** 2018-06-22

**Authors:** Lidya Salim, Chris McKim, Jean-Paul Desaulniers

**Affiliations:** University of Ontario Institute of Technology, Faculty of Science 2000 Simcoe Street North Oshawa ON L1H 7K4 Canada Jean-Paul.Desaulniers@uoit.ca

## Abstract

The use of short interfering RNAs (siRNAs) as therapeutics holds great promise, but chemical modifications must first be employed to improve their pharmacokinetic properties. This study evaluates the *in vitro* cellular uptake and knock-down efficacy of cholesterol-modified triazole-linked siRNAs targeting firefly luciferase in the absence of a transfection carrier. These siRNAs displayed low cytotoxicity and excellent dose-dependent knockdown in HeLa cells in the 500 to 3000 nM concentration range, with a 70–80% reduction in firefly luciferase activity. Our results indicate that this modification is compatible with the RNA interference pathway, and is less cytotoxic and more effective than a commercially-available triethylene glycol (TEG) cholesterol modification.

## Abstract 2

The use of short interfering RNAs (siRNAs) as therapeutics holds great promise, but chemical modifications must first be employed to improve their pharmacokinetic properties.

## Graphical abstract

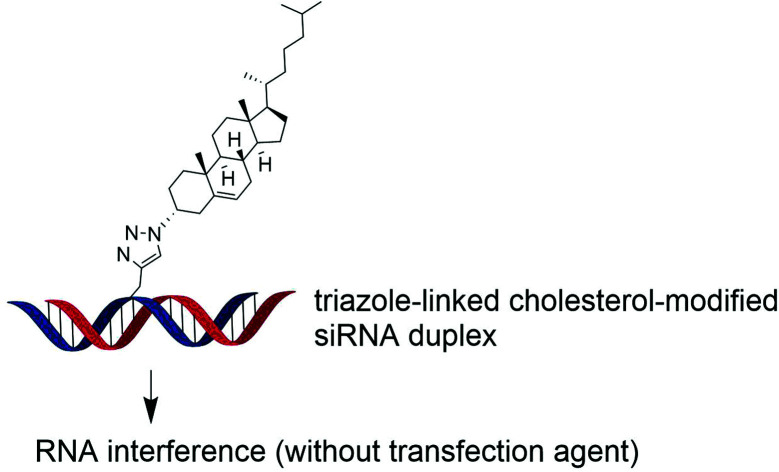

RNA interference (RNAi) is an endogenous pathway that utilizes double-stranded RNA to suppress translation, resulting in sequence-specific gene silencing.^1^ The initial step involves cleavage of long double-stranded RNA into smaller 21–23 nucleotide fragments, termed short interfering RNAs (siRNAs), which are incorporated into the RNA-induced silencing complex (RISC).^2^ RISC unwinds and dissociates the duplex, retaining the antisense strand which is used as a guiding sequence to recognize and degrade complementary mRNA.^2,3^ Since many diseases are characterized by aberrant gene expression, the use of siRNAs as therapeutics holds great promise.^4,5^ Unfortunately, there are some limitations associated with the structure of siRNAs, including low stability, poor cellular uptake and off-target effects, which must be addressed in order to harness the full potential of RNAi therapeutics.^6,7^ Although several chemical modifications have been employed to improve the pharmacological properties of siRNAs, there is still no universal modification able to simultaneously improve all of these limitations.^8,9^

Due to their large size and anionic backbone, siRNAs have difficulties crossing cellular membranes. Therefore, several delivery systems and carriers have been investigated, including viral vectors, cationic polymers and liposomes.^10–13^ Another strategy involves direct conjugation of siRNAs to small molecules such as GalNac or hydrophobic molecules to enhance cellular uptake.^14^ Cholesterol is a hydrophobic biomolecule and a key component of cellular membranes, as it helps maintain their integrity.^15^ Various cholesterol-conjugated drugs and anticancer agents have been studied and have demonstrated enhanced pharmacokinetic profiles, bioavailability and delivery.^16,17^ Cholesterol modifications have also been successful at increasing siRNA lipophilicity and improving cellular uptake without the need of transfection carriers.^18–20^

Recently, we reported a straightforward synthesis of a cholesterol phosphoramidite, bound covalently to a spacer *via* a triazole linkage.^21^ This cholesterol-bearing spacer was then incorporated within the central region of the siRNA sense strand through solid-phase RNA synthesis.^21^ Our biological studies in HeLa cells showed that these siRNAs were able to downregulate exogenous firefly luciferase mRNA in a dose-dependent manner using the transfection carrier Lipofectamine® 2000. In this study, we further investigate the biological activity and gene-silencing efficacy of these siRNAs in the absence of a transfection carrier. Fig. 1 compares the structure of native RNA with our cholesterol-modified triazole-linked spacer (X) and a commercially-available 3′-end triethylene glycol cholesterol (Chol-TEG) modification.

**Fig. 1:**
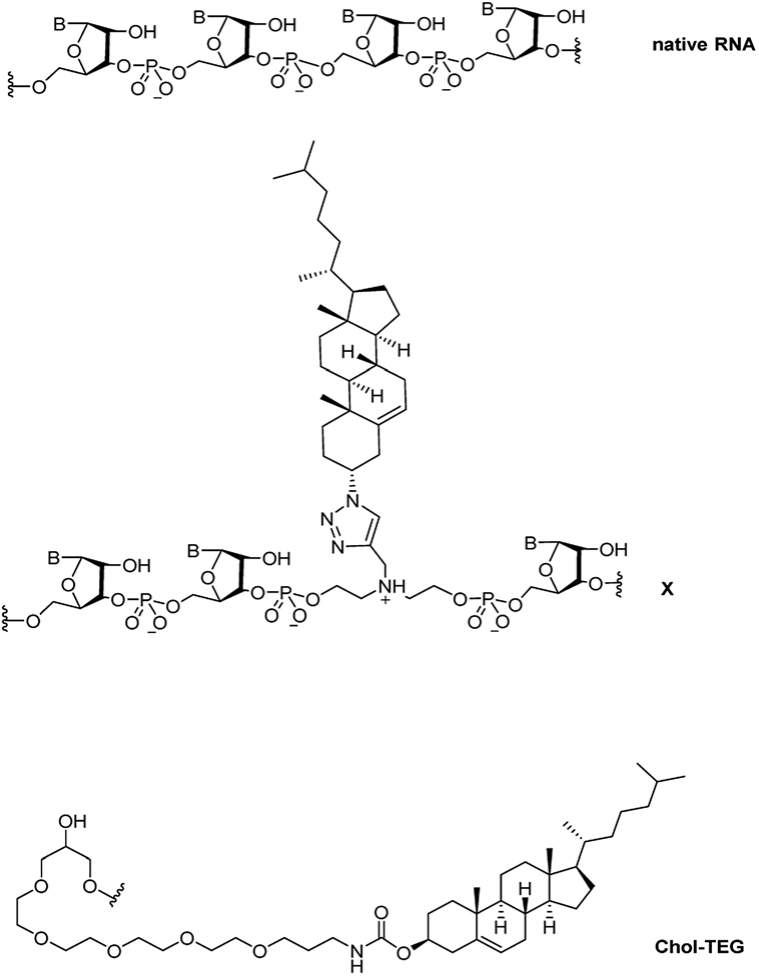
Structural differences between native RNA, cholesterol-modified triazole-linked spacer (X) and the commercially-available 3′-end cholesterol triethylene glycol (Chol-TEG) modification.

To examine the silencing potential of these siRNAs, HeLa cells were co-transfected with plasmids coding for firefly luciferase (target) and *Renilla* luciferase (internal control) respectively. After a 4 hour incubation period, culture media was discarded and cells were washed twice with phosphate-buffer saline to remove any traces of Lipofectamine® 2000. Fresh media was added to each well, followed by addition of the respective siRNA treatment with concentrations ranging from 1 to 3000 nM. After an additional 16 hour incubation period, cells were lysed and the gene-silencing efficacy of siRNAs was assessed using the dual-luciferase reporter gene assay. All siRNAs target firefly luciferase and their sequences are highlighted in Table 1. siRNAs X1 and X2 contain the triazole-linked cholesterol modification within the central region of the sense strand (positions 9 and 10 from the 5′-end, respectively). siRNA X5 contains the triazole-linked cholesterol modification at the 3′-end of the sense strand. Chol-TEG contains the commercially-available 3′-end triethylene glycol cholesterol derivative.

**Table 1:** Sequences of anti-luciferase siRNA and *T*_m_ data^a^

RNA	siRNA duplex	*T*_m_	Δ*T*_m_
wt	5′-CUUACGCUGAGUACUUCGAtt-3′	72.7	—
	3′-ttGAAUGCGACUCAUGAAGCU-5′		
X1	5′-CUUACGCU***X***AGUACUUCGAtt-3′	61.6	−11.1
	3′-ttGAAUGCGACUCAUGAAGCU-5′		
X2	5′-CUUACGCUG***X***GUACUUCGAtt-3′	62.5	−10.2
	3′-ttGAAUGCGACUCAUGAAGCU-5′		
X5	5′-CUUACGCUGAGUACUUCGA***X***t-3′	69.8	−2.9
	3′-ttGAAUGCGACUCAUGAAGCU-5′		
Chol-TEG	5′-CUUACGCUGAGUACUUCGAtt***Ch***-3′	65.3	−6.7
	3′-ttGAAUGCGACUCAUGAAGCU-5′		

a ***X*** corresponds to the single triazole-linked cholesterol modification. ***Ch*** corresponds to the commercial triethylene glycol modification. The top strand corresponds to the sense strand; the bottom strand corresponds to the antisense strand. In all duplexes, the 5′-end of the bottom antisense strand contains a phosphate group.

To first ensure that the siRNAs used in this study were effective in silencing firefly luciferase, a gene-silencing assay was conducted using Lipofectamine® 2000 as a transfection carrier. These siRNAs show effective gene-silencing activity in a dose-dependent manner at low concentrations (8 to 800 pM) (Fig. S1 in ESI†). In a carrier-free protocol, as observed in Fig. 2, the cholesterol-modified triazole-linked siRNAs (X1, X2, and X5) exhibit potent gene silencing, with 70–80% reduction in firefly luciferase activity in the 500 to 3000 nM concentration range. As previously reported, placing a chemical modification within the central region of the sense strand may impact thermal destabilization,^22–24^ however, this does not seem to alter gene-silencing efficacy. In fact, the IC_50_s for these thermally-destabilized centrally-modified siRNAs X1 and X2 were 243.6 nM and 307.1 nM respectively. The 3′-modified siRNA X5 also exhibited effective gene silencing, with an IC_50_ of 189.2 nM. Previous studies have reported that the 3′-end of the sense strand is able to accommodate bulky groups.^25^

**Fig. 2:**
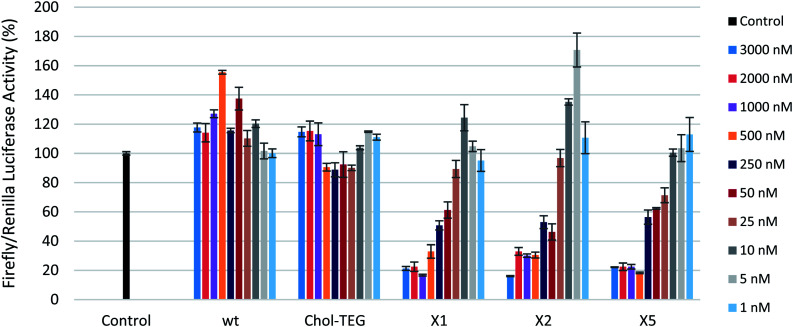
Reduction in firefly luciferase expression in HeLa cells as a function of siRNA activity ranging from 1 to 3000 nM in the absence of a transfection carrier. Firefly luciferase expression was normalized to *Renilla* luciferase.

The wild-type siRNA (wt), which lacks a cholesterol modification, did not display any gene-silencing activity in our carrier-free study. This was expected as unmodified siRNAs are known to have difficulties in crossing the cellular membrane unassisted. The use of 3′-end cholesterol modifications has been reported in the literature with varying degrees of success.^18,26,27^ As such, we decided to investigate the gene-silencing efficacy of a commercially-available 3′-end triethylene glycol (TEG) cholesterol modification (Chol-TEG) using our carrier-free transfection protocol as a comparison to our cholesterol-modified triazole-linked siRNAs (X1, X2, and X5). Interestingly, the Chol-TEG siRNAs displayed poor gene-silencing abilities in the entire range between 1 to 3000 nM.

It is not entirely clear why the cholesterol-modified triazole-linked siRNAs (X1, X2 and X5) exhibit potent gene silencing compared to the siRNA Chol-TEG. One possibility is that the conformationally constrained triazole functionality in some way is benefiting the siRNA. Furthermore, the nitrogen atom used to functionalize the molecule with the triazole-cholesterol group is positive under physiological pH, which may also assist in cellular uptake. In contrast, the Chol-TEG group contains a neutral, polar and flexible polyethylene linker, which may poorly impact the overall cellular uptake profile of the siRNA.

In order to determine the toxicological effect of siRNA treatments, an XTT cell proliferation assay was performed. The XTT reagent is reduced by mitochondrial succinic dehydrogenase in metabolically-active cells to a highly-pigmented formazan derivative. The absorbance of this product can be quantified and used to assess cellular viability. As seen in Fig. 3, siRNAs bearing the X spacer (siRNAs X1, X2 and X5) cause minimal toxicity even at high concentrations. HeLa cells treated with 3000 nM wt siRNA show a 20–30% decrease in viability compared to cells treated with our cholesterol-modified siRNAs. In addition, high concentrations (1000–3000 nM) of Chol-TEG siRNA imparted high cytotoxicity, causing a 60–80% reduction in cell viability, perhaps explaining why these siRNAs did not display successful gene-silencing activity. It is unclear why siRNAs X1, X2 and X5 are the least toxic compared to wt and Chol-TEG. However, some studies have identified that molecules functionalized with triazoles are non-toxic.^28,29^ Thus, it is possible that the triazole functionality reduces the cytotoxicity of siRNAs.

**Fig. 3:**
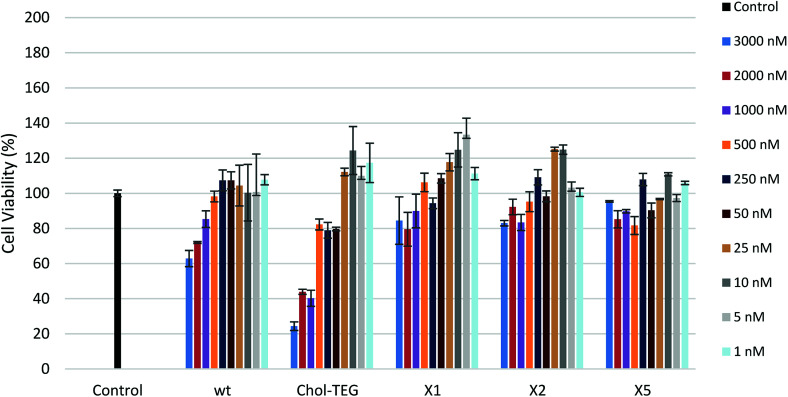
HeLa cell viability after treatment with various siRNA concentrations (1–3000 nM) using the XTT Cell Proliferation Assay.

## Conclusions

In conclusion, cholesterol-modified triazole-linked siRNAs show excellent dose-dependent gene silencing of exogenous firefly luciferase mRNA in the absence of a transfection carrier. These results indicate that our modification is compatible with the RNA interference pathway when placed at both the central region and 3′-end of the sense strand of siRNAs. This could provide a novel approach to improve cellular uptake, and perhaps assist with other downstream applications such as packaging of liposomes and lipid-nanoparticles.

## Conflicts of interest

There are no conflicts to declare.

## Supplementary Material

RA-008-C8RA03908A-s001
