# Supplementary material for: Effective carrier-free gene-silencing activity of cholesterol-modified siRNAs
Source: RSC Adv. 2018 Jun 22;8(41):22963–6. doi: 10.1039/c8ra03908a (PMC9081628; doi:10.1039/c8ra03908a)
Supplement: RA-008-C8RA03908A-s001 [file RA-008-C8RA03908A-s001.pdf]

Electronic Supplementary Material (ESI) for RSC Advances.
This journal is © The Royal Society of Chemistry 2018

Supporting Information

Effective Carrier-Free Gene-Silencing Activity of Cholesterol-Modified siRNAs

Lidya Salim, Chris McKim and Jean-Paul Desaulniers

Faculty of Science, University of Ontario Institute of Technology, 2000 Simcoe Street North, Oshawa, Ontario L1H 7K4 Canada

Jean-Paul.Desaulniers@uoit.ca

Table of Contents

Experimental Nucleic Acid and Biological Procedures

General....S2
Procedure for Characterizing Oligonucleotides through ESI Q-TOF....S2
Procedure for HPLC Characterization....S2
Procedure for Sub-Culturing of HeLa Cells (Passaging)....S2
Procedure for *in vitro* Dual-Reporter Luciferase Assay in the Presence of a Transfection Reagent....S2
Procedure for *in vitro* Dual-Reporter Luciferase Assay in the Absence of a Transfection Reagent....S3
Procedure for XTT Cellular Proliferation Assay....S3

Figures and Tables

Table S1: Predicted and recorded mass of chemically-modified RNAs....S4
Figure S1: siRNA Gene-Silencing of luciferase with Lipofectamine 2000®....S4
Figure S2. HPLC chromatogram of X1 siRNA....S5
Figure S3. HPLC chromatogram of X2 siRNA....S6
Figure S4. HPLC chromatogram of X5 siRNA....S6

Experimental Nucleic Acid and Biological Procedures

General

Unless otherwise stated, all starting reagents were obtained from commercial sources without additional purification. Antisense strand RNA and CHOL-TEG RNA sequences were purchased from and characterized by IDTDNA. Equimolar amounts of sense and antisense RNAs were incubated at 95°C for 2 min in a binding buffer (75.0 mM KCl, 50.0 mM Tris-HCl, 3.00 mM MgCl2, pH 8.30). This solution was cooled slowly to room temperature, allowing the siRNAs to anneal.

Procedure for Characterizing Oligonucleotides through ESI Q-TOF

All single-stranded RNAs (ssX1, ssX2, and ssX5) were gradient eluted through a Zorbax Extend C18 HPLC column with a MeOH/H2O (5 : 95) solution containing 200 mM hexafluoroisopropyl alcohol and 8.1 mM triethylamine, and finally with 70% MeOH. The eluted RNAs were subjected to ESI-MS (ES-), producing raw spectra of multiply-charged anions and through resolved isotope deconvolution, the molecular weights of the resultant neutral oligonucleotides were confirmed. The final neutral mass of the RNAs were confirmed using this method.

Procedure for HPLC Characterization

High-performance liquid chromatography (HPLC) using a C18 4.6 mm x 150 mm reverse phase column on a Waters 1525 Binary HPLC Pump with a Waters 2489 UV/Visible Detector, eluting from 5% to 95% acetonitrile in 0.1 M triethylammonium acetate (TEAA) buffer (pH: 7). Spectra were processed using the Empower 3 software.

Sub-Culturing of HeLa Cells (Passaging)

Biological assays were performed using human epithelial cervix carcinoma cells (HeLa cells). Cells were maintained in Dulbecco's Modified Eagle's Medium (DMEM), supplemented with 10% fetal bovine serum (FBS) and 1% penicillin-streptomycin (Sigma), and incubated at 37°C with 5% CO_2. Once they reached 80-90% confluency, cells were passaged and diluted to a concentration of $1x10^6$ cells/mL. To continue the cell line, 1 mL of this was added to a new cell culture flask containing 24 mL DMEM (10% FBS, 1% penicillin-streptomycin).

Procedure for *in vitro* Dual-Reporter Luciferase Assay in the Presence of a Transfection Reagent

Prior to transfection, HeLa cells were seeded on 12-well plates (Falcon®) containing 1 mL DMEM (10% FBS, 1% penicillin-streptomycin) at a density of 100,000 cells per well. Cells were incubated at 37 ^{0}C with 5% CO_2 for 24 hours until they reached 90% confluence. Then, varying concentrations of anti-luciferase siRNAs (8, 80 and 800 pM) were co-transfected with both pGL3 and pRLSV40 luciferase-expressing plasmids using Lipofectamine 2000® (Invitrogen) in 1X Gibco's Opti-Mem Reduced Serum according to the manufacturer's protocol. Cells were incubated for an additional 24 hours at 37 ^{0}C in 5% CO_2. The medium was discarded, cells were

washed twice with 1X phosphate buffered saline (PBS) and lysed with 1X passive lysis buffer (Promega) over a 20-minute period at room temperature. Cell lysates were loaded onto white and opaque 96-well plates (Costar) in triplicate. Using the Dual-Luciferase® Reporter Kit (Promega), Lar II and Stop & Glo® substrates were added to the cell lysates and enzymatic activity of firefly and *Renilla* luciferase vectors were measured independently using a Synergy HT (Bio-Tek) plate luminometer. The ratio of firefly/*Renilla* luminescence expressed as a percentage relates the reduction in firefly expression to siRNA efficacy when compared to untreated controls. Each data point represents the average of at least two independent assays, each with three technical replicates, with the indicated error (SDOM). The IC50 values were determined with Prism using the variable slope model when the log(inhibitor) was plotted against normalized expression.

Procedure for *in vitro* Dual-Reporter Luciferase Assay in the Absence of a Transfection Reagent

Prior to transfection, HeLa cells were seeded on 24-well plates (Falcon®) containing 350 µL DMEM (10% FBS, 1% penicillin-streptomycin) at a density of 50,000 cells per well. Cells were incubated at 37 ^{0}C with 5% CO_2 for 24 hours until they reached 90% confluence. Then, cells were co-transfected with both pGL3 and pRLSV40 luciferase-expressing plasmids using Lipofectamine 2000® (Invitrogen) in 1X Gibco's Opti-Mem Reduced Serum according to the manufacturer's protocol. Cells were incubated for 4 hours at 37 ^{0}C in 5% CO_2 after which the growth medium was discarded and each well was washed twice with 1 mL of 1X PBS to ensure that no transfection reagent remained in solution. 50 µL DMEM (10% FBS, 1% penicillin-streptomycin) was then added to each well. Anti-luciferase siRNA treatments were prepared by adding 1 µL of the respective siRNA to 20 µL 1X Gibco's Opti-Mem Reduced Serum. Each treatment was added to the respective well, at concentrations of 1, 5, 10, 25, 50, 250, 500, 1000, 2000 and 3000 nM. Cells were incubated for an additional 16 hours at 37 ^{0}C in 5% CO_2 before cell lysing. Luciferase activity was assessed as described above. Each value is the average of at least 3 different experiments with the indicated error (SDOM). The IC50 values were determined with Prism using the variable slope model when the log(inhibitor) was plotted against normalized expression.

Procedure for XTT Cellular Proliferation Assay

Cellular viability after siRNA treatment was determined using the XTT Cell Proliferation Assay Kit (ATCC®). Prior to transfection, HeLa cells were seeded on 96-well plates (Falcon®) containing 150 µL DMEM (10% FBS, 1% penicillin-streptomycin) at a density of 2,500 cells per well. Cells were incubated at 37 °C in 5% CO_2 for 24 hours, after which they were co-transfected with pGL3 and pRLSV40 luciferase-expressing plasmids and incubated for an additional 4 hours, as previously described. After this incubation period, the growth medium was discarded and each well was washed twice with 250 µL 1X PBS. Cells were then treated in triplicate with varying concentrations (1, 5, 10, 25, 50, 250, 500, 1000, 2000 and 3000 nM) of each siRNA and then incubated for an additional 24 hours at 37 °C with 5% CO_2 before treatment with 50 µL of XTT, activated with 2% *N*-methyl dibenzopyrazine methyl sulfate. Cells were incubated for 2 hours at 37 °C with 5% CO_2. Absorbance was measured at 475 nm and 660 nm using a Synergy HT (Bio-Tek) microplate reader. Specific absorbance was calculated: A_{475nm} (experimental) – A_{475nm} (Blank) – A_{660nm} (experimental). Results were normalized to an untreated control.

Figures and Tables

Table S1: Predicted and recorded masses for chemically-modified RNAs

Sample Number	Sense RNAs	Predicted Neutral Mass	Observed Neutral Mass
**ssX1**	5'- CUUACGCU***X***AGUACUUCGAtt -3'	6958.6	6959.2
**ssX2**	5'- CUUACGCUG***X***GUACUUCGAtt -3'	6955.2	6953.2
**ssX5**	5'- CUUACGCUGAGUACUUCGA***X***t -3'	6998.6	6998.8

ESI Q-TOF were recorded in a negative electrospray mode after HPLC elution using two mobile phases; MeOH/H_2O 5:95 (v/v) with 200 mM hexafluoroisopropyl alcohol and 8.1 mM triethylamine, and 70% MeOH. ***X*** corresponds to the single triazole-linked cholesterol modification. The top strand corresponds to the sense strand; the bottom strand corresponds to the antisense strand. In all duplexes, the 5′-end of the bottom antisense strand contains a phosphate group.

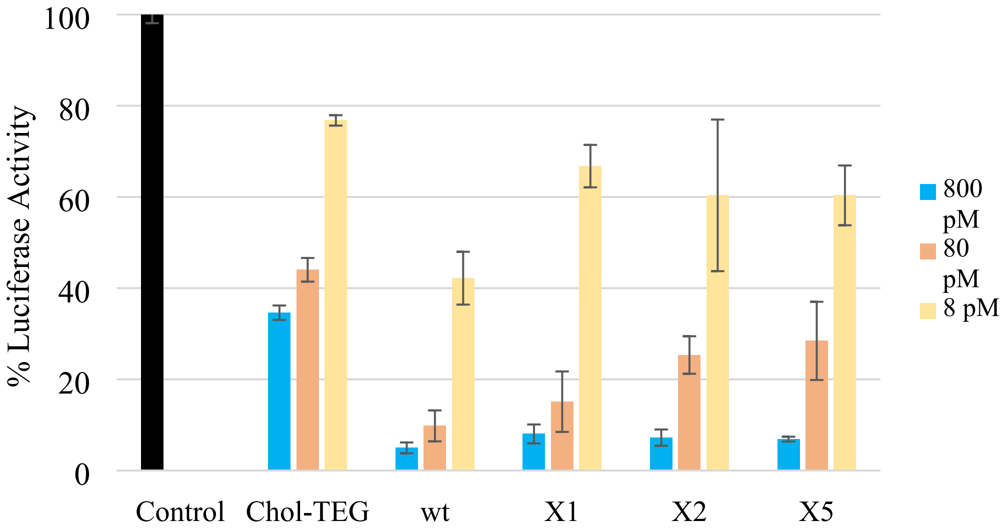

Figure S1. Reduction in firefly luciferase activity as a function of siRNA activity. The commercially-available 3’-end triethylene glycol cholesterol siRNA (**Chol-TEG**), **wt** siRNA and triazole-linked cholesterol siRNAs (**X1**, **X2** and **X5**) were tested in HeLa cells at 8, 80 and 800 pM in the presence of a transfection reagent, with firefly luciferase expression normalized to *Renilla* luciferase.

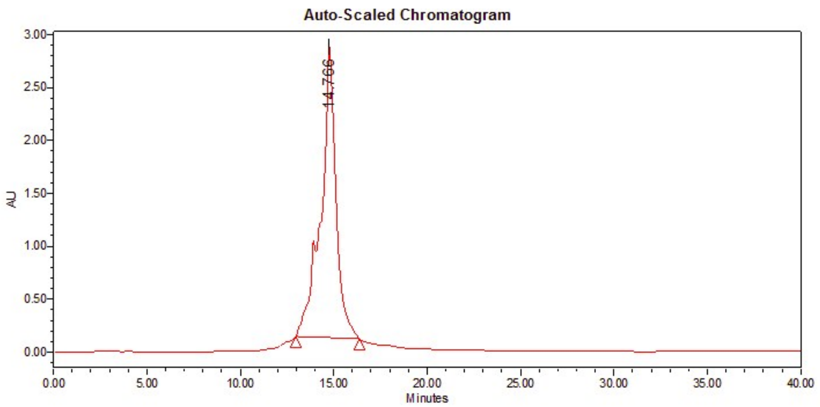

Figure S2. HPLC chromatogram of X1 siRNA, eluting from 5% to 100% acetonitrile in 0.1 M TEAA buffer over 40 min.

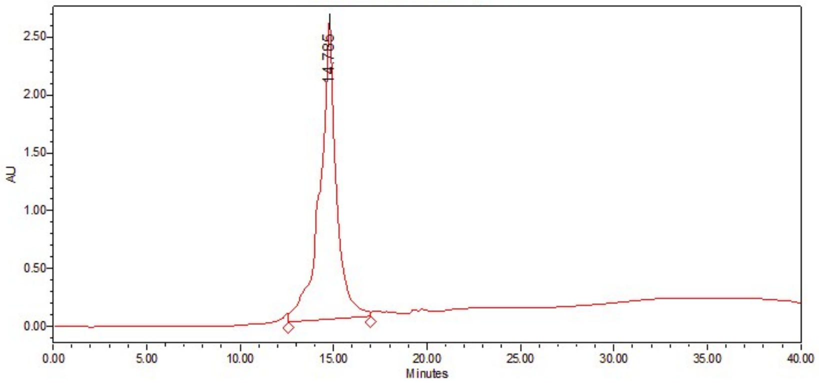

Figure S3. HPLC chromatogram of X2 siRNA, eluting from 5% to 100% acetonitrile in 0.1 M TEAA buffer over 40 min.

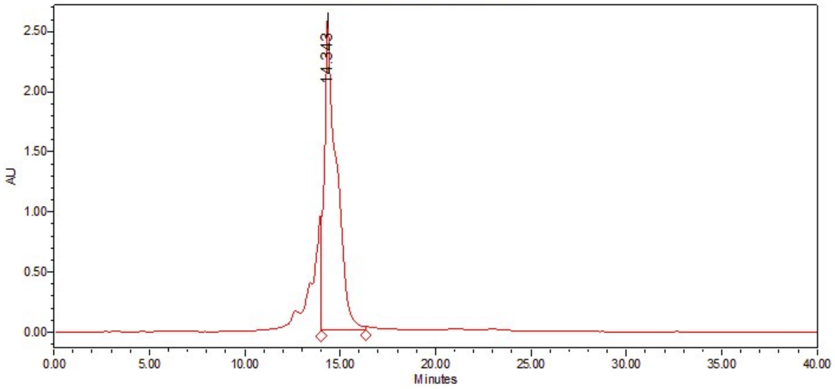

Figure S4. HPLC chromatogram of X5 siRNA, eluting from 5% to 100% acetonitrile in 0.1 M TEAA buffer over 40 min.
